# Endothelial Colony-Forming Cells Derived From Pregnancies Complicated by Intrauterine Growth Restriction Are Fewer and Have Reduced Vasculogenic Capacity

**DOI:** 10.1210/jc.2013-2580

**Published:** 2013-10-08

**Authors:** Peter I. Sipos, Stephane L. Bourque, Carl A. Hubel, Philip N. Baker, Colin P. Sibley, Sandra T. Davidge, Ian P. Crocker

**Affiliations:** Maternal and Fetal Health Research Centre (P.I.S., C.P.S., I.P.C.), Manchester Academic Health Science Centre, University of Manchester, Central Manchester University Hospitals National Health Service Foundation Trust, Manchester M13 9WL, United Kingdom; Department of Obstetrics and Gynecology (S.L.B.), University of Alberta, Edmonton, Alberta, Canada T6G 2S2; and Magee-Womens Research Institute and Department of Obstetrics, Gynecology, and Reproductive Sciences (C.A.H.), University of Pittsburgh, Pittsburgh, Pennsylvania 15213

## Abstract

**Context:**

Endothelial colony-forming cells (ECFCs) are the only putative endothelial progenitor cells capable of vasculogenesis, and their dysfunction may represent a risk factor for cardiovascular disease. Intrauterine growth restriction (IUGR) is a pregnancy-related disorder associated with long-term cardiovascular risk.

**Objective:**

Our objective was to determine whether ECFCs derived from pregnancies complicated by IUGR exhibit altered vasculogenic potential.

**Design and Setting:**

This was a prospective cohort study; patients were recruited at St. Mary's Hospital, Manchester, United Kingdom.

**Participants:**

Twenty-three women with normal pregnancies and 13 women with IUGR-complicated pregnancies at gestational ages above 37 weeks were included.

**Main Outcome Measures:**

Vasculogenic capacity of rigorously characterized ECFCs was investigated in vivo by measuring blood vessel formation in collagen/fibronectin gels implanted in mice; proliferative, migratory, and chemotactic abilities were assessed in cell culture. Placental uptake of fetal ECFCs, assessed by differences in arterial and venous cord blood content, was determined by flow cytometry.

**Results:**

In vivo, IUGR ECFCs formed fewer blood vessels (*P* < .001) and capillaries (*P* = .001) compared with normal pregnancy-derived ECFCs. In culture conditions, IUGR ECFCs had reduced proliferation (*P* = .01) and migration (*P* = .007) and diminished chemotactic abilities to stromal cell-derived factor 1 (*P* = .007) coupled with reduced hypoxia-induced matrix metalloproteinase-2 release (*P* = .02). Finally, in IUGR pregnancies, the number of ECFCs was lower in arterial cord blood (*P* = .002) and placental uptake of cells was reduced (*P* < .001).

**Conclusions:**

ECFCs derived from IUGR cord blood are rarefied and dysfunctional, resulting in diminished vasculogenic potential; this could be a cause of placental dysfunction in IUGR, with long-term postnatal implications for cardiovascular function in offspring.

Endothelial colony-forming cells (ECFCs) are a well-defined homogeneous cell population phenotypically identical to mature endothelial cells in most respects, but are distinct functionally because they are capable of extensive proliferation (single cells can give rise to hundreds of progeny cells) and neointima formation (1), properties of true endothelial progenitor cells (2). As such, ECFCs are actively involved in vasculogenesis and vascularization by directly populating the new endothelial surface. Acting in concert with ECFCs are circulating progenitor cells (CPCs), a heterogeneous subpopulation of hematopoietic lineage, which promote neovascularization principally by modifying the extracellular matrix (via production of matrix metalloproteinases [MMPs]), attracting ECFCs and orchestrating their migration via secretion of paracrine factors (eg, stromal cell-derived factor 1 [SDF-1], vascular endothelial growth factor [VEGF]-B, and IL-8) (reviewed in Ref. 3).

Intrauterine growth restriction (IUGR) is a complication of pregnancy in which the fetus fails to achieve its genetically determined growth potential (4). Although IUGR is a condition with complex etiologies, suboptimal placental vascularization, and hence diminished blood and nutrient delivery, is often implicated (5–7). Recently, we have shown that fetal-derived ECFCs play a physiological role in vasculogenesis within the placenta (unpublished data) and the pregnant uterus (8). We therefore reasoned that ECFCs derived from pregnancies complicated by IUGR would exhibit altered proliferative and vasculogenic capacity. To test this hypothesis, we examined cord blood-derived ECFC numbers and ability to form blood vessels in vivo as well as growth characteristics, migration, chemotaxis, and MMP-2 production in culture.

## Patients and Methods

### Patients

Women with normal (n = 23) and IUGR-complicated pregnancies (n = 13) at gestational ages above 37 weeks were recruited at St Mary's Hospital, Manchester, United Kingdom, between October 2008 and December 2010. IUGR was defined as a term neonate with an individualized birth weight ratio less than 5 (9, 10). Exclusion criteria included medical or obstetric complications of pregnancy other than IUGR, use of any medication, or adverse medical history. For flow cytometry, >500 μL of arterial and venous umbilical blood samples were collected using 20-gauge hypodermic needles (Brown) from double-clamped umbilical cords immediately after delivery. For cell culture, >15 mL mixed cord blood was collected from unclamped sections of cord. Blood was collected into Vacutainer tubes containing EDTA as anticoagulant (Becton Dickinson). Samples for flow cytometry were kept on ice, whereas samples for culture were maintained at room temperature with continuous shaking. Consent was obtained from all participants, and the study was approved by the North West Research Ethics Committee, United Kingdom, and the Ethics Committee of Central Manchester Foundation Trust Hospitals, United Kingdom.

### ECFC isolation, culture, and phenotyping

Mononuclear cells were isolated and expanded from whole fetal blood according to established protocols (11). Briefly, whole blood was diluted (3:4 with PBS) and subjected to density gradient centrifugation (711*g* for 30 minutes) using Histopaque-1077 (Sigma-Aldrich Co). The mononuclear cell layer was then collected and placed into collagen-I–coated dishes (60 million cells per well in a 6-well cell culture plate) using fully supplemented EGM-2 media (Lonza Group Ltd). Adherent single layers of cobblestone-shaped, late outgrowth cells are suggestive of ECFCs. The identity of isolated cells was confirmed by a range of stringent phenotype criteria and extensive functional assays as previously described (P. I. Sipos, X. Fan, S. L. Bourque, J. L. Stanley, I. J. Andersson, A. L. Ridgway, M. Wareing, C. A. Hubel, P. N. Baker, S. T. Davidge, C. P. Sibley, I. P. Crocker, unpublished data). Briefly, ECFCs were morphologically characterized by assessing expression of endothelial markers CD31, CD34, CD144, CD105, CD146, *Ulex europaeus* lectin, and hematopoietic markers CD45 or CD14 using immunostaining and flow cytometry. Functionally, uptake of acetylated low-density lipoprotein as well as the ability to form tubules in basement membrane matrix (Matrigel; BD Biosciences) were assessed in vitro. Capacity to form colonies and repopulate wells after single cell sorting (using a FacsAria III Cell Sorter; BD Biosciences) was also assessed. Finally, vasculogenic capacity, a hallmark feature of ECFC, was assessed by implanting collagen/fibronectin gels populated with isolated cells subfascially in NOD/SCID mice, and de novo vessel formation was assessed (see below for details); a subset of ECFCs were retrovirally transduced to express enhanced green fluorescent protein as a means of distinguishing between implanted human cells and those of murine origin.

### In vivo vasculogenesis bioassay

Experiments involving mice were approved by the University of Alberta Health Sciences Animal Policy and Welfare Committee in accordance with the Canadian Council on Animal Care guidelines. Artificial collagen-based plugs were prepared according to established procedures (11). Briefly, 1 × 10^6^ ECFCs and 2.5 × 10^5^ adipose-derived stem cells (Invitrogen), grown separately, were detached from culture and suspended in a collagen gel matrix consisting of a (1:1) mix of collagen-1 (BD Biosciences) and fibronectin (Fisher Scientific Ltd) in a total volume of 500 μL reagent mix (HEPES, sodium bicarbonate, fetal bovine serum, and endothelial basal medium-2 [pH 7.4]). Once gelled in a 24-well culture plate, the cell-containing block was covered with EGM-2 media and incubated overnight. The resulting contracted gels (approximately 2–3 mm in diameter) were sc implanted into the flanks of female adult (18–20 weeks of age) virgin immunodeficient NOD/SCID mice (strain 005557; The Jackson Laboratory) under isoflurane inhalation anesthesia (induction, 5%; maintenance, 1.5% in oxygen) through a parasagittal skin incision in the lumbar area. Pairs of artificial tissue blocks containing ECFCs from growth-restricted pregnancies and normal pregnancies were bilaterally implanted into each animal. Once the wound was closed with silk sutures, pain relief was provided with sc ketoprofen (5 mg/kg), and the mice were treated with Augmentin (amoxicillin/clavulanate) in their drinking water to prevent postoperative infections. After 14 days, the implants were harvested by dissection under a stereomicroscope after isoflurane euthanasia, cleaned of extraneous connective tissue, and fixed in 4% zinc formalin overnight. Dissected implants were then embedded in paraffin and cut as 5-μm sections at increments of approximately 50 to 70 μm such that 10 sections represented the entire tissue block. Tissue sections were stained with hematoxylin and eosin. Three nonconsecutive sections were examined under light microscopy (Eclipse 80i; Nikon) and assessed for small and large vessels, with a minimum of 6 fields per slide at both ×10 and ×60. Numbers and surface areas of cross-sections of complex vascular structures were measured at ×10, whereas numbers of capillaries were assesses at ×60 magnification, using ImageJ image analysis software (National Institutes of Health). Capillaries were defined as tubular structures with a diameter less than 7 μm, formed by the vacuolization of not more than 1 endothelial cell; vessels were defined as more complex structures wider than 7 μm and formed by more than 1 ECFC.

### ECFC proliferation studies

In the setting of primary cultures of ECFCs, colonies represent the clones of individual progenitor cells. Colonies are defined as discrete clusters consisting of more than 50 cells; culture plates were monitored daily for their appearance. The numbers of colonies, the time elapsed between seeding and first appearance of visible colonies, and population doubling times (12) as determined by live cell count during the first 3 passages were recorded.

### Cell migration and chemotaxis assays

For migration assays, ECFCs were detached from culture, suspended in phenol red-free and serum-free DMEM (Invitrogen). Cells (3 × 10^5^) were seeded over the porous membrane of a 24-well fluorescent cell migration assay kit (Millipore), and phenol red-free and serum-free DMEM was added to the lower chamber. For chemotaxis assays, the lower chamber was supplemented with chemotactic factors: SDF-1α (20 ng/mL), IL-8 (100 ng/mL), or VEGFβ (2 μg/mL). Plates for migration and chemotaxis assays were incubated in a humidified environment at 5% CO_2_ for 24 hours. Chambers were handled and stained according to manufacturer's recommendations, and microplates were read on a SpectraMax Gemini XS plate reader (Molecular Devices) and data analyzed using Soft Max Pro version 4.8 software (Molecular Devices). Numbers of migrated cells were calculated from fluorescence intensities using a standard curve. Experiments were carried out in duplicate.

### Gelatin zymography for MMP-2 activity

MMP-2 activity was assessed by gelatin zymography. Conditioned medium (endothelial basal medium-2 -incubated for 12 hours with cells and then spun at 12 000*g* to remove cell debris) from ECFCs were loaded on 8% SDS-PAGE gels copolymerized with gelatin (2 mg/mL, type A, from porcine skin; Sigma Aldrich). After 1 hour electrophoresis, gels were washed with 2.5% Triton X-100 at room temperature for 1 hour, during which time the solution was changed every 20 minutes. Gels were then incubated for 18 hours at 37°C in incubation buffer: 50 mmol/L Tris-HCl, 150 mmol/L NaCl, 5 mmol/L CaCl_2_, and 0.05% NaN_3_ (pH 7.6). After incubation, gels were stained with 0.05% Coomassie Brilliant Blue (Sigma Aldrich) in a mixture of methanol/acetic acid/water (2.5:1:6.5 vol/vol) and destained in aqueous 4% methanol/8% acetic acid (vol/vol). Gelatinolytic activities were detected as transparent bands against a dark blue background. Digital images were obtained with a high-resolution scanner (Expression 1680; Epson), and band intensities were quantified using Quantity One version 4.3.1 software (Bio-Rad Laboratories Inc). Bands were normalized to lysate protein concentration. For protein lysates, confluent monolayers of cells were washed with PBS, and each well of a 6-well plate was treated with 400 μL/well of cell lysis buffer (25mM Tris, 0.5% Triton X-100), supplemented with Halt Protease Inhibitor Cocktail (Thermo Fisher Scientific Inc). Plates were scraped and centrifuged at 12 000*g* to remove residual cell debris. Protein content was assayed using the Pierce BCA Protein Assay (Thermo Fisher) according to the manufacturer's instructions.

### Hypoxic treatments

Cells were treated with EGM-2 media that was preincubated in hypoxic conditions (1% O_2_) for 24 hours before experimentation. Cells were incubated in media in an oxygen-controlled (21% O_2_ or 1% O_2_) incubator for 72 hours. After incubation, cells were removed, rinsed with PBS, and used for gelatin zymography, as described above.

### Flow cytometry

Flow cytometry was used to calculate the quantity of both subsets of endothelial progenitor cells (EPC) sequestered by the examined placentas, by determining the circulating ECFC and CPC numbers in both the umbilical arteries and corresponding veins as percentages of mononuclear cells and calculating the placental uptake. Venous and arterial umbilical blood samples were obtained from the umbilical cord at birth of the placenta. After blockage of nonspecific binding sites with 20 μL FcR blocking reagent (Miltenyi Biotec), conjugated antibodies were added to 125 μL of tested blood directly in optimized quantities: 4 μL anti-CD31-fluorescein isothiocyanate (BD Biosciences), 5 μL anti-CD133-R-phycoerythrin (Miltenyi Biotec), 5 μL anti-KDR-phycoerythrin (R&D Systems), 10 μL anti-CD45-allophycocyanin-H7 (BD Biosciences), 3 μL anti-CD34-allophycocyanin (BD Biosciences), lysed in nonfixing lysis buffer (Pharmalyse lysis Buffer (BD Biosciences). Before running on a CyAn flow cytometer (DAKO), 5 μL of the viability stain 7-aminoactinomycin D (7-AAD) (BD Biosciences) was added. Flow cytometer was calibrated using Sphero Rainbow fluorescent particles (BD Biosciences), whereas gain, compensation, and autofluorescence were accounted for by measurements with corresponding isotype controls. Data were analyzed by Summit Flow Cytometry Analysis Software (DAKO), and ECFCs were determined in the mononuclear gate and characterized by the discriminators 7AAD^−^/CD31^bright^/CD45^−^/KDR^+^/CD34^+^, whereas CPCs were determined as 7-AAD^−^/CD31^−^/CD45^+^/CD133^+^/CD34^+^ (13) (see Supplemental Figure 1, published on The Endocrine Society's Journals Online website at http://jcem.endojournals.org).

### Statistical analyses

Data are presented as medians and interquartile ranges. Distribution was examined with D'Agostino-Pearson and Kolmogorov-Smirnov test, and compared by Student's *t* test, Mann-Whitney *U* test, or ANOVA with Bonferroni post hoc test, as appropriate. EPC numbers in arterial and venous cord blood were analyzed by two-way ANOVA with Bonferroni post hoc test. Data were analyzed with the Prism version 5 software package (GraphPad Software Inc).

## Results

### Patient demographics

There were no differences between groups in gestational age, maternal body mass index, smoking status, or ethnicity (Table 1). Mothers of neonates in the IUGR group were younger and were more frequently primigravidas.

**Table 1.** Patient Information at Time of Delivery

Parameter	Control	IUGR	*P* Value
n	23	13	
Individualized birth ratio	66.7 ± 20.3	1.5 ± 2.2	<.001
Birth weight, g	3503 ± 341	2237 ± 535	<.01
Placental weight, g	661 ± 119	363 ± 98	<.05
Gestational age, wk	39.5 ± 1.5	38.1 ± 2.2	NS
Maternal BMI, kg/m^2^	27.1 ± 8.6	26.1 ± 4.3	NS
Parity, n	0.87 ± 0.8	0.12 ± .35	<.05
Maternal age, y	30.5 ± 6.1	23.5 ± 2.7	<.05
Smoker, %	6.2	11.1	NS
Cesarean section, %	50	68	NS

Abbreviation: BMI, body mass index; NS, not significant.

### Characterization of outgrowth colonies and cells used for further studies

With current culture techniques, ECFCs emerge as late outgrowth colonies after plating cells in the density gradient layer corresponding to mononuclear leukocytes, whereas other cell types die (11). Thorough morphological and functional characterizations of ECFCs were performed. Briefly, isolated ECFCs displayed a cobblestone morphology, expressed several endothelial markers (CD31, CD34, CD144, CD105, CD146, and *U. europaeus* lectin), and were devoid of any hematopoietic (CD45) or mononuclear (CD14) markers. In vitro, these cells were capable of acetylated low-density lipoprotein uptake and formed tubes in basement membrane matrix (Matrigel; BD Biosciences). Importantly, after single cell sorting (using a FacsAria III Cell Sorter; BD Biosciences), ECFCs were capable of forming colonies and repopulating entire wells, whereas similarly treated differentiated endothelial cells (human umbilical vein endothelial cells) were not. Finally, ECFCs, but not human umbilical vein endothelial cells, were shown to display vasculogenic properties by de novo vessel formation within subfascial murine implantations of artificial tissue blocks, thereby confirming their role in neovascularization.

### ECFCs from growth-restricted offspring have diminished vasculogenic capacity in vivo

**Figure 1.**
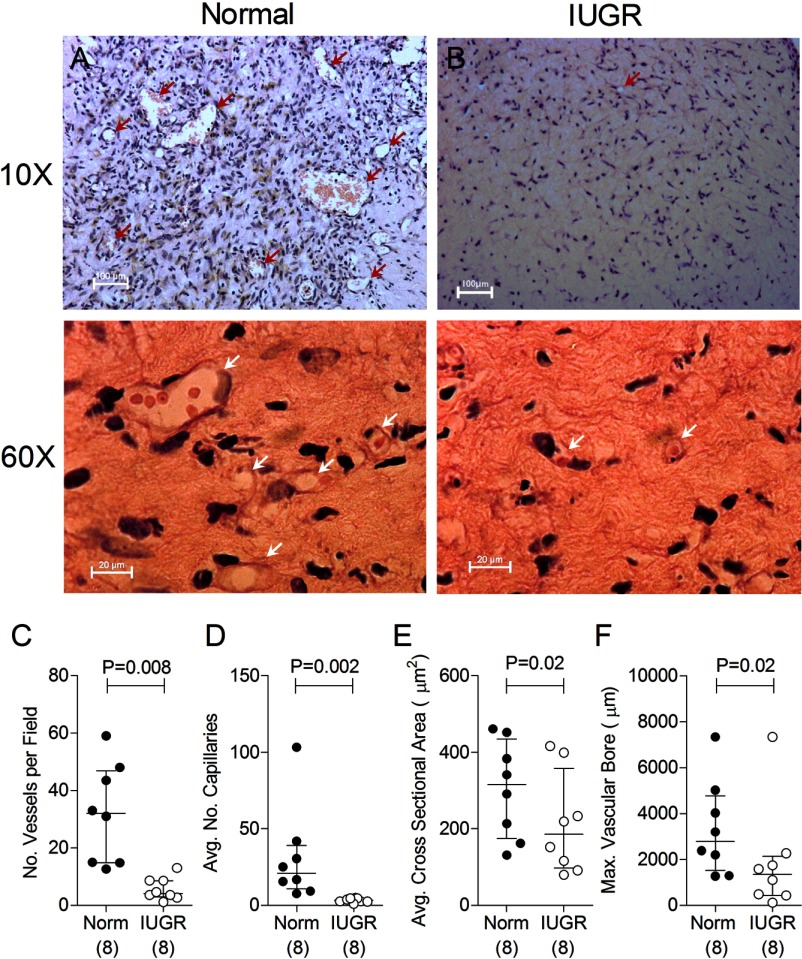
IUGR-derived fetal ECFCs have reduced vasculogenic capacity. ECFCs expanded from cord blood obtained from normal (Norm) and IUGR-complicated pregnancies were suspended in collagen-fibronectin gels with human adult adipose-derived stem cells and implanted subfascially into adult NOD/SCID mice. A and B, Representative images of collagen-fibronectin implants stained with hematoxylin and eosin showing vascular structures (arrows) formed by ECFCs derived from cord blood of normal (A) and IUGR-complicated (B) pregnancies. Images in the upper panels are at ×10 magnification (scale bar, 100 μm) to show complex vascular structures; images in the lower panels are at ×60 magnification to show simple capillaries (scale bar, 20 μm). C–F, Comparisons of quantity of complex vascular structures per microscopic view (C), numbers of vacuoles representing newly formed capillaries (D), total vascularized surface per microscopic view (E), and largest vessel bore observed (F). Numbers in parentheses show the number of independent cord blood samples tested.

As discussed above, the only circulating cell type known to possess vasculogenic abilities is ECFC (3). Therefore, to best assess the function of fetal ECFCs obtained from normal and IUGR-complicated pregnancies, we performed an in vivo vasculogenesis bioassay in mice. ECFC-driven blood vessel formation in implants populated with IUGR-derived ECFCs was markedly impaired. Specifically, compared with controls (Figure 1A), IUGR ECFC-populated implants had 8-fold fewer large blood vessels (defined as those consisting of complex multicellular make-up, Figure 1B; data summarized in Figure 1C), and had 6-fold fewer capillaries (Figure 1D) formed de novo. Compared with controls, vessels formed by ECFCs from IUGR pregnancies had 2-fold narrower bores, expressed as an average and maximal cross-sectional area of the vascular lumen (Figure 1, E and F).

### ECFCs of IUGR offspring have impaired proliferation, migration, and MMP-2 production

**Figure 2.**
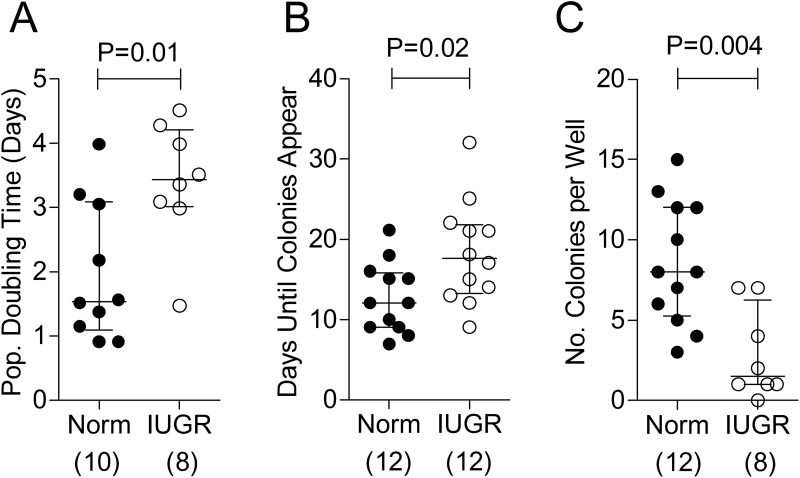
ECFCs obtained from pregnancies complicated by IUGR have reduced proliferation in culture. A and B, Proliferative capacity was assessed by average calculated population (Pop.) doubling time (A) and the minimum number of days before colonies were identified in culture (B); see Patients and Methods for details. C, The number of ECFCs identified in culture after plating 6 × 10^7^ mononuclear cells after 14 days. Numbers in parentheses show the number of independent cord blood samples tested. Abbreviation: Norm, normal.

A high rate of proliferation, as a primary determinant of the quantity of differentiated cells produced, is a quintessential feature of endothelial progenitor function. Indeed, proliferation influences the capacity of ECFCs to populate the neointimal surface and repair damaged endothelium (1). We assessed proliferation via 2 methods. First, growth characteristics of ECFCs (derived from 6 × 10^7^ mononuclear cells of cord blood) were observed in primary culture. The time elapsed from plating the mononuclear cells to the appearance of the first (fully characterized) ECFC colonies, assessed by daily observations, was longer in the case of ECFCs from IUGR-complicated pregnancies (Figure 2A). Second, the population doubling time of ECFCs, assessed by live cell counts during the first 2 to 3 passages, was 2.3 times longer in IUGR cases (Figure 2B). Furthermore, the number of ECFC colonies that appeared in primary culture before confluence (a reflection of the concentration of viable ECFCs) was fewer from IUGR compared with control samples (Figure 2C). Taken together, these observations are indicative of a lower proliferative rate of ECFCs derived from growth-restricted neonates.

**Figure 3.**
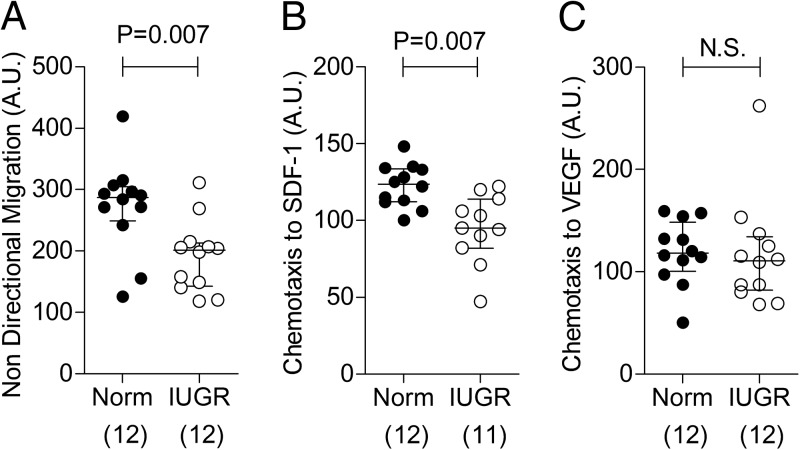
ECFCs obtained from pregnancies complicated by IUGR have reduced migration and chemotactic properties in culture. A–C, Nondirectional migration (A) and chemotaxis in response to SDF-1α (20 ng/mL) (B) and VEGFβ (2 μg/mL) (C) in culture conditions. Numbers in parentheses show the number of independent cord blood samples tested. Abbreviations: A.U., arbitrary units; Norm, normal; N.S., not significant.

Migration of progenitor cells to the site of nascent vessel formation is crucial for vasculogenesis. Nondirectional migration was found to be lower in IUGR-derived ECFCs (Figure 3A), and chemotaxis in response to SDF-1 was significantly attenuated (Figure 3B). Migratory responses to VEGF-B (Figure 3C) and IL-8 (data not shown) were unaltered.

**Figure 4.**
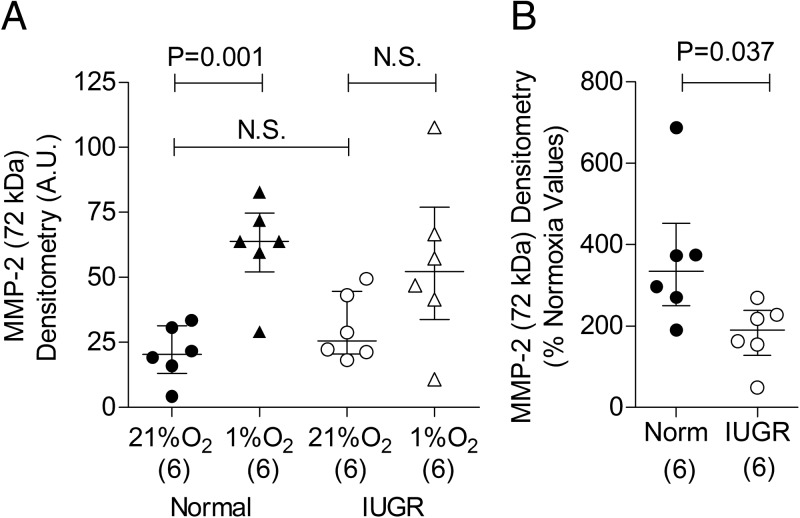
ECFCs obtained from pregnancies complicated by IUGR have altered MMP-2 secretory profiles. A, MMP-2 release by cultured ECFCs after 72 hours incubation in normoxia (21% O_2_) and hypoxia (1% O_2_). B, Effect of hypoxia on MMP-2 release shown as a percentage of normoxic values. Numbers in parentheses show the number of independent cord blood samples tested. Abbreviations: A.U., arbitrary units; Norm, normal; N.S., not significant.

MMP-2 release from ECFCs was also assessed (Figure 4) because of its role in degrading extracellular matrix components and enabling cell migration (14, 15). Under normal normoxic conditions, MMP-2 release from IUGR-derived ECFCs was not different from normal cells (Figure 4A). Hypoxia, an important stimulus for endothelial cell migration, caused increased MMP-2 release in normal ECFCs but not in IUGR-derived ECFCs (Figure 4A); a direct comparison between pregnancy groups confirmed that the hypoxia-mediated increase in MMP-2 release was greater in normal compared with IUGR-derived ECFCs (Figure 4B).

### ECFCs from IUGR-derived cord blood are rare

**Figure 5.**
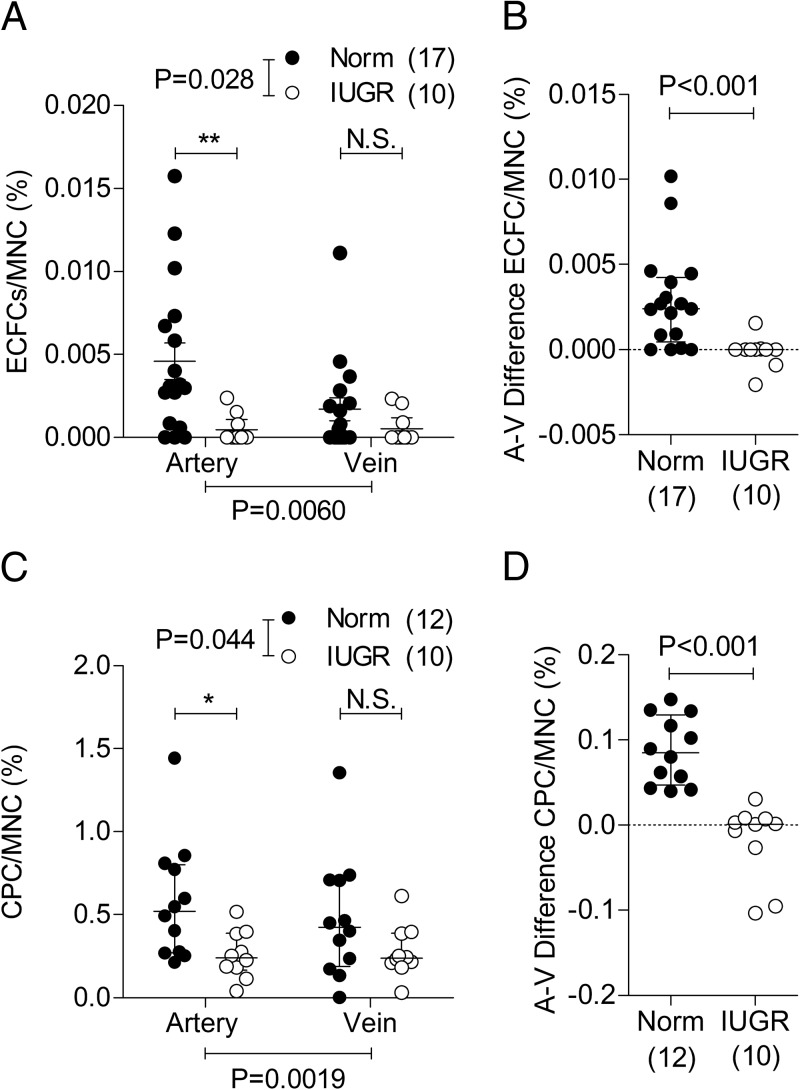
A and C, ECFCs (A) and CPCs (C) are fewer in cord blood obtained from IUGR-complicated pregnancies. B and D, Calculated arteriovenous (A-V) differences of ECFCs (B) and CPCs (D). Values are expressed as a percentage of total blood mononuclear cells (MNC). Numbers in parentheses show the number of independent cord blood samples tested. Abbreviations: Norm, normal; N.S., not significant.

Although altered numbers are unlikely to account for the diminished vasculogenesis observed in the implant studies (Figure 1), because gels from both pregnancy groups consisted of equal numbers of ECFCs, a regional reduction of EPCs could represent an additional mechanism of defective placental vasculogenesis in situ. We therefore sought to determine whether ECFC numbers in cord blood and their placental uptake were changed in pregnancies complicated with IUGR. Using flow cytometry, ECFCs were more abundant in umbilical arterial blood than venous cord blood (*P* = .0060 for overall effect by two-way ANOVA), but this difference was evident only in normal pregnancies (*P* < .001) and not in IUGR pregnancies (*P* > .05) (Figure 5A). Cord blood from IUGR-complicated pregnancies had fewer ECFCs compared with normal pregnancies (*P* = .028 for overall effect), and this was evident in arterial but not in venous cord blood. By extension, the net difference between arterial and venous ECFC content, taken as an indication of the number of EPCs sequestered by the placenta, was markedly decreased in IUGR pregnancies compared with controls (Figure 5B). Consistent with these observations, circulating CPCs (the main driving force of ECFC migration) were higher in arterial compared with venous cord blood (*P* = .002 for overall effect), and again, this effect was evident only in normal pregnancies (*P* < .001; Figure 5C). CPCs in umbilical cord blood of IUGR fetuses were also lower overall (*P* = .044), and again, differences were observed only in arterial but not venous blood, corresponding to a smaller arterial-venous gradient in growth-restricted cases (Figure 5D).

## Discussion

In the present study, we have shown several differences in ECFC function and numbers, including vasculogenic capacity as well as proliferative, migratory, and chemotactic ability between those cells isolated from cord blood of normal pregnancies and those complicated by IUGR. Importantly, we excluded known pregnancy-associated complications known to contribute to ECFC dysfunction, such as gestational diabetes (16), and preterm birth (17). To our knowledge, this is the first study to demonstrate alterations in ECFC function in pregnancies strictly characterized as isolated IUGR. IUGR is a pregnancy complication with many etiologies; therefore, multiple mechanisms may underlie the observed ECFC dysfunction. We have identified several cellular changes that could explain, at least in part, the reduced ECFC vasculogenic capacity associated with IUGR.

The finding that ECFCs from IUGR pregnancies have reduced proliferative capacity is potentially important, because vascular formation to the extent observed in the control collagen/fibronectin implants undoubtedly requires extensive ECFC proliferation. The number of IUGR ECFCs populating the collagen matrices would be lower than controls after a few days, thereby limiting vascular formation. Diminished nondirectional migratory and chemotactic behavior may also be indicative of diminished vasculogenic capacity. SDF-1 plays a particularly important role in EPC homing to areas of vessel formation as well as cell polarity and morphology, thereby influencing migration (18). Consequently, reduced responsiveness to this chemokine may impede the capacity of IUGR ECFCs to migrate and orient appropriately for vascular formation.

Hypoxia increased MMP-2 secretion in our control ECFCs, consistent with the notion that MMPs are important in mediating the tissue responses to hypoxia, including neovascularization (14). Recently, MMP-2 has been shown to be indispensable for EPC-mediated vessel formation (19). The failure of IUGR-derived ECFCs to increase release of MMP-2 under hypoxic conditions may therefore represent an important mechanism underlying the diminished vasculogenesis observed by these cells.

The observation that ECFCs and CPCs were higher in arterial compared with venous cord blood is consistent with the notion that these cells are taken up by the placenta from the fetal circulation. We demonstrate that numbers of ECFCs in arterial cord blood are decreased in IUGR-complicated pregnancies, suggesting fewer cells locally available for vasculogenesis within the placenta. Similarly decreased numbers of CPCs, the main migratory driving force and stimulant of ECFCs, in arterial cord blood not only confirms these findings but also suggests a potentially less stimulated state of IUGR ECFCs when in the placenta. Although lower arterial-venous cell gradients indicate diminished ECFC and CPC delivery to the placenta in IUGR pregnancies, it is not presently clear whether this deficit stems from an impaired ability of the IUGR placenta to sequester cells or whether alterations in progenitor cell survival, proliferation, adhesion, and mobilization are largely responsible. Based on the observations that there are very few circulating ECFCs and CPCs in arterial cord blood from IUGR pregnancies, and we found marked differences in the proliferative and migratory phenotype of these cells in culture conditions, it is tempting to speculate that intrinsic deficiencies in progenitor cell function are at least partly responsible.

An interesting question that arises from the present work is whether the altered EPC dysfunction could represent a mechanistic link between early adverse conditions and long-term health consequences. Indeed, IUGR fetuses are predisposed to chronic illness in later life, including cardiovascular disease (20, 21). However, the mechanisms linking an abnormal intrauterine environment to long-term cardiovascular complications, including endothelial dysfunction and vascular damage, remain elusive. Because ECFCs contribute to vascular repair and their dysfunction is a risk factor for cardiovascular decline (22–25), reduced circulating numbers of ECFCs, as well as reduced vasculogenic capacity of these cells, may provide a tentative link between IUGR and increased susceptibility to cardiovascular disease. Indeed, numbers and function of ECFCs have been shown to be reduced in conditions associated with cardiovascular disease (26, 27). In addition, it has been proposed that endothelial injury in the absence of adequate numbers of CPCs may contribute to the progression of disease (28); it therefore stands to reason that diminished proliferation and diminished vasculogenic capacity of ECFCs associated with IUGR could also hinder vascular repair during adult life. However, it is not yet known whether ECFC proliferation and vasculogenic capacity remain impaired throughout life; future studies are needed to address these issues.

In summary, fetal ECFCs are dysfunctional and are rare in pregnancies affected by IUGR. Consequently, their contribution to placental vasculogenesis is suboptimal. This may prove to be important in understanding the cause of placental dysfunction in IUGR and the link between small size at birth and programming of CVD in later life, given the role of ECFCs in endothelial repair in adults.

## References

[1] Ingram DA Mead LE Tanaka H. Identification of a novel hierarchy of endothelial progenitor cells using human peripheral and umbilical cord blood. Blood. 2004;104(9):2752–2760 15226175 10.1182/blood-2004-04-1396

[2] Lin Y Weisdorf DJ Solovey A Hebbel RP. Origins of circulating endothelial cells and endothelial outgrowth from blood. J Clin Invest. 2000;105(1):71–77 10619863 10.1172/JCI8071 PMC382587

[3] Sipos PI Crocker IP Hubel CA Baker PN. Endothelial progenitor cells: Their potential in the placental vasculature and related complications. Placenta. 2010;31(1):1–10 19917514 10.1016/j.placenta.2009.10.006

[4] Romo A Carceller R Tobajas J. Intrauterine growth retardation (IUGR): Epidemiology and etiology. Pediatr Endocrinol Rev. 2009;6(Suppl 3):332–336 19404231

[5] Chen CP Bajoria R Aplin JD. Decreased vascularization and cell proliferation in placentas of intrauterine growth-restricted fetuses with abnormal umbilical artery flow velocity waveforms. Am J Obstet Gynecol. 2002;187(3):764–769 12237661 10.1067/mob.2002.125243

[6] Mayhew TM Ohadike C Baker PN Crocker IP Mitchell C Ong SS. Stereological investigation of placental morphology in pregnancies complicated by pre-eclampsia with and without intrauterine growth restriction. Placenta. 2003;24(2–3):219–226 12566249 10.1053/plac.2002.0900

[7] Kiserud T Ebbing C Kessler J Rasmussen S. Fetal cardiac output, distribution to the placenta and impact of placental compromise. Ultrasound Obstet Gynecol. 2006;28(2):126–136 16826560 10.1002/uog.2832

[8] Sipos PI Rens W Schlecht H. Uterine vasculature remodelling in human pregnancy involves functional macro-chimerism by endothelial colony forming cells of fetal origin. Stem Cells. 2013;31(7):1363–1370 23554274 10.1002/stem.1385 PMC3813980

[9] Sanderson DA Wilcox MA Johnson IR. The individualised birthweight ratio: A new method of identifying intrauterine growth retardation. Br J Obstet Gynaecol. 1994;101(4):310–314 8199077 10.1111/j.1471-0528.1994.tb13616.x

[10] Wilcox MA Johnson IR Maynard PV Smith SJ Chilvers CE. The individualised birthweight ratio: A more logical outcome measure of pregnancy than birthweight alone. Br J Obstet Gynaecol. 1993;100(4):342–347 8494835 10.1111/j.1471-0528.1993.tb12977.x

[11] Mead LE Prater D Yoder MC Ingram DA. Isolation and characterization of endothelial progenitor cells from human blood. Curr Protoc Stem Cell Biol. 2008;Chapter 2:Unit 2C.1 10.1002/9780470151808.sc02c01s6 18770637

[12] Melero-Martin JM Bischoff J. Chapter 13. An in vivo experimental model for postnatal vasculogenesis. Methods Enzymol. 2008;445:303–329 19022065 10.1016/S0076-6879(08)03013-9

[13] Duda DG Cohen KS Scadden DT Jain RK. A protocol for phenotypic detection and enumeration of circulating endothelial cells and circulating progenitor cells in human blood. Nat Protoc. 2007;2(4):805–810 17446880 10.1038/nprot.2007.111 PMC2686125

[14] Cheng XW Kuzuya M Nakamura K. Mechanisms underlying the impairment of ischemia-induced neovascularization in matrix metalloproteinase 2-deficient mice. Circ Res. 2007;100(6):904–913 17322177 10.1161/01.RES.0000260801.12916.b5

[15] Basire A Sabatier F Ravet S. High urokinase expression contributes to the angiogenic properties of endothelial cells derived from circulating progenitors. Thromb Haemost. 2006;95(4):678–688 16601839

[16] Ingram DA Lien IZ Mead LE. In vitro hyperglycemia or a diabetic intrauterine environment reduces neonatal endothelial colony-forming cell numbers and function. Diabetes. 2008;57(3):724–731 18086900 10.2337/db07-1507

[17] Ligi I Simoncini S Tellier E. A switch toward angiostatic gene expression impairs the angiogenic properties of endothelial progenitor cells in low birth weight preterm infants. Blood 2011;118(6):1699–1709 21659549 10.1182/blood-2010-12-325142

[18] Shen L Gao Y Qian J. The role of SDF-1alpha/Rac pathway in the regulation of endothelial progenitor cell polarity; homing and expression of Rac1, Rac2 during endothelial repair. Mol Cell Biochem. 2012;365(1–2):1–7 21964561 10.1007/s11010-011-1083-z

[19] Wu Y Dai J Schmuckler NG. Cleaved high molecular weight kininogen inhibits tube formation of endothelial progenitor cells via suppression of matrix metalloproteinase 2. J Thromb Haemost. 2010;8(1):185–193 19874467 10.1111/j.1538-7836.2009.03662.x PMC3142619

[20] Barker DJ Eriksson JG Forsén T Osmond C. Fetal origins of adult disease: Strength of effects and biological basis. Int J Epidemiol. 2002;31(6):1235–1239 12540728 10.1093/ije/31.6.1235

[21] Rueda-Clausen CF Morton JS Davidge ST. The early origins of cardiovascular health and disease: who, when, and how. Semin Reprod Med. 2011;29(3):197–210 21710396 10.1055/s-0031-1275520

[22] Werner N Kosiol S Schiegl T. Circulating endothelial progenitor cells and cardiovascular outcomes. N Engl J Med. 2005;353(10):999–1007 16148285 10.1056/NEJMoa043814

[23] Huang L Hou D Thompson MA. Acute myocardial infarction in swine rapidly and selectively releases highly proliferative endothelial colony forming cells (ECFCs) into circulation. Cell Transplant. 2007;16(9):887–897 18293887 10.3727/096368907783338181

[24] Jiga J Hoinoiu B Stoichitoiu T. Induction of therapeutic neoangiogenesis using in vitro-generated endothelial colony-forming cells: an autologous transplantation model in rat. J Surg Res. 2013;181(2):359–368 22818979 10.1016/j.jss.2012.06.059

[25] Schwarz TM Leicht SF Radic T. Vascular incorporation of endothelial colony-forming cells is essential for functional recovery of murine ischemic tissue following cell therapy. Arterioscler Thromb Vasc Biol. 2012;32(2):e13–e21 22199368 10.1161/ATVBAHA.111.239822

[26] Campioni D Zauli G Gambetti S. In vitro characterization of circulating endothelial progenitor cells isolated from patients with acute coronary syndrome. PLoS One. 2013;8(2):e56377 23409178 10.1371/journal.pone.0056377 PMC3569417

[27] Meneveau N Deschaseaux F Séronde MF. Presence of endothelial colony-forming cells is associated with reduced microvascular obstruction limiting infarct size and left ventricular remodelling in patients with acute myocardial infarction. Basic Res Cardiol. 2011;106(6):1397–1410 21904841 10.1007/s00395-011-0220-x

[28] Hill JM Zalos G Halcox JP. Circulating endothelial progenitor cells, vascular function, and cardiovascular risk. N Engl J Med. 2003;348(7):593–600 12584367 10.1056/NEJMoa022287

